# Idarucizumab for a traumatic head injury patient taking dabigatran

**DOI:** 10.1186/s12245-018-0202-y

**Published:** 2018-10-03

**Authors:** Shuhei Maruyama, Koichi Hayakawa, Shuji Kanayama, Hiromu Iwamura, Daiki Wada, Fukuki Saito, Yasushi Nakamori, Yasuyuki Kuwagata

**Affiliations:** 1 Department of Emergency and Critical Care Medicine, Kansai Medical University Medical Center, 10-15 Fumizono-cho, Moriguchi, Osaka, 570-8507 Japan; 2 Department of Emergency and Critical Care Medicine, Kansai Medical University Hospital, 2-3-1 Shinmachi, Hirakata, Osaka, 573-1191 Japan

**Keywords:** Dabigatran, Direct oral anticoagulants, Idarucizumab, Specific neutralizing drug, Traumatic brain injury, Traumatic intracranial hemorrhage

## Abstract

**Background:**

Dabigatran is one of the four drugs currently used as a direct oral anticoagulant in Japan. Idarucizumab, which specifically targets dabigatran, was recently approved in Japan. We present a case of intracranial hemorrhage in a traumatic brain injury patient taking dabigatran who was treated by administering idarucizumab.

**Case presentation:**

A 72-year-old man was injured in a traffic accident and was transferred to our emergency room. On arrival, his Glasgow Coma Scale score was 14 (eye, 3; verbal, 5; motor, 6), and his other vital signs were stable. Computed tomography (CT) imaging on arrival showed a small intracranial hematoma. A second CT 3 h later revealed expansion of the hematoma. We received information that he was taking dabigatran only after the second CT. Idarucizumab was then promptly administered, and emergency craniotomy for hematoma removal was performed. There was no tendency for bleeding during the operation, and blood transfusion was not required during the perioperative period. Although the patient underwent additional surgery for subdural effusion and hydrocephalus, his postoperative course was uneventful. He was transferred to a rehabilitation hospital on postoperative day 102.

**Conclusion:**

We managed a patient taking dabigatran who suffered traumatic intracranial hemorrhage by administering idarucizumab preoperatively without the need for blood transfusion perioperatively. We suggest that idarucizumab could be a potent therapeutic bridge to definitive surgical management in such patients with traumatic brain injury who are taking dabigatran.

## Background

As the population ages, more people are likely to be on anticoagulant drugs due to the higher prevalence of atrial fibrillation or valvular surgery [1, 2]. Direct oral anticoagulants (DOAC) have a favorable benefit-risk profile compared with vitamin K antagonists and are associated with similar or better outcomes in cases of major bleeding and urgent procedures [3–5]. A study of intracranial bleeding due to blunt trauma in patients taking anticoagulants showed that the mortality and surgical intervention rates were significantly lower in the group receiving DOAC than in the warfarin group [6]. Acute traumatic coagulopathy can sometimes occur in head trauma patients, thus making it difficult to complete the surgery and highlighting the importance of the careful management of the coagulation system [7].

Idarucizumab is a humanized monoclonal antibody fragment that binds dabigatran with high affinity and specificity and rapidly reverses its anticoagulant activity. We present the case of a patient with severe traumatic brain injury taking dabigatran whom we treated with the administration of idarucizumab and craniotomy for hematoma removal.

## Case presentation

A 72-year-old man was injured when the bicycle he was riding collided with a car (we estimated that the time of injury was 4 h after the last taking of dabigatran). On hospital arrival, his Glasgow Coma Scale (GCS) score was 14 (eyes, 3; verbal, 5; motor, 6), and his vital signs were stable.

**Fig. 1:**
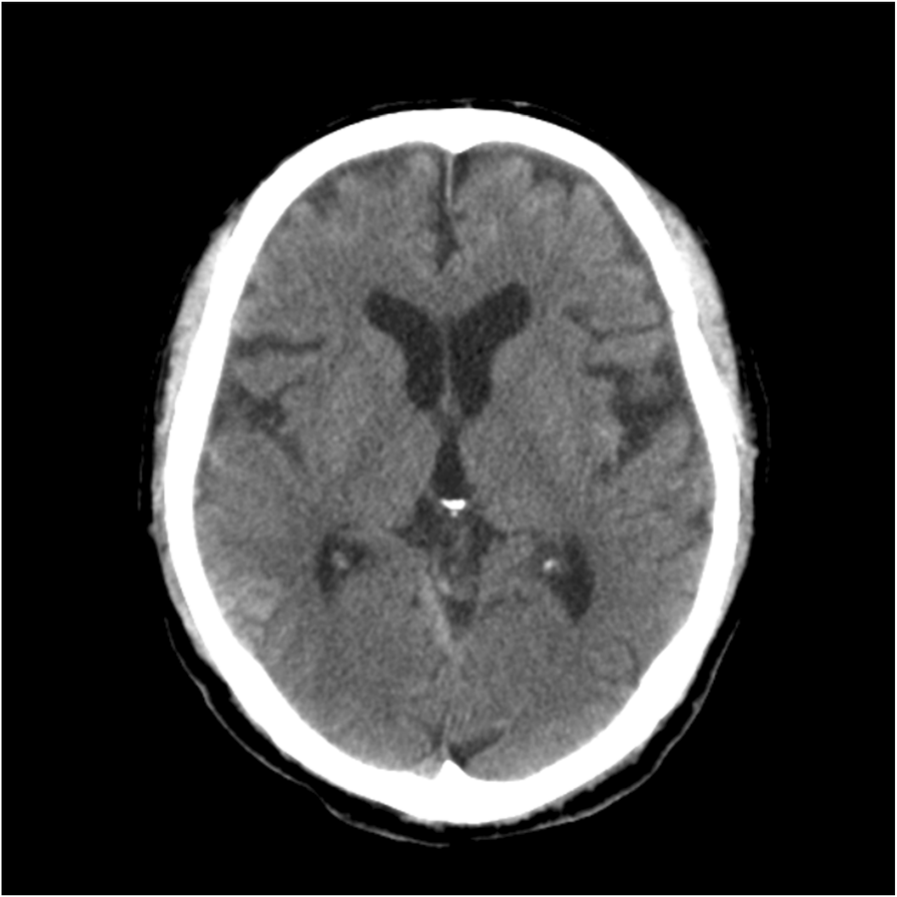
Head CT on admission showed a right temporal lobe contusion and subdural hematoma in the cerebellum tent

Arterial blood gas analysis results while receiving oxygen by reservoir mask at a rate of 8 L/min and blood test findings are shown in Table 1. Whole-body computed tomography (CT) showed a right temporal lobe contusion, acute subdural hematoma, zygomatic bone fracture, and third lumbar compression fracture. A representative head CT image is shown in Fig. 1.

**Table 1:** Results of arterial blood gas analysis and blood tests on arrival

Arterial blood gas findings		Blood test findings	
pH	7.462	PT	30%
PaO_2_	242 mmHg	aPTT	94.9 s
PaCO_2_	33.7 mmHg	FDP D-dimer	12.8 μg/mL
Base excess	0.4 mmol/L	Hb	12.3 g/dL
Lactate	13 mg/dL	Platelets	199 × 10^3^/μL
		Blood urea nitrogen	24 mg/dL
		Serum creatinine	1.2 mg/dL
		Creatinine clearance	47 mL/min

**Fig. 2:**
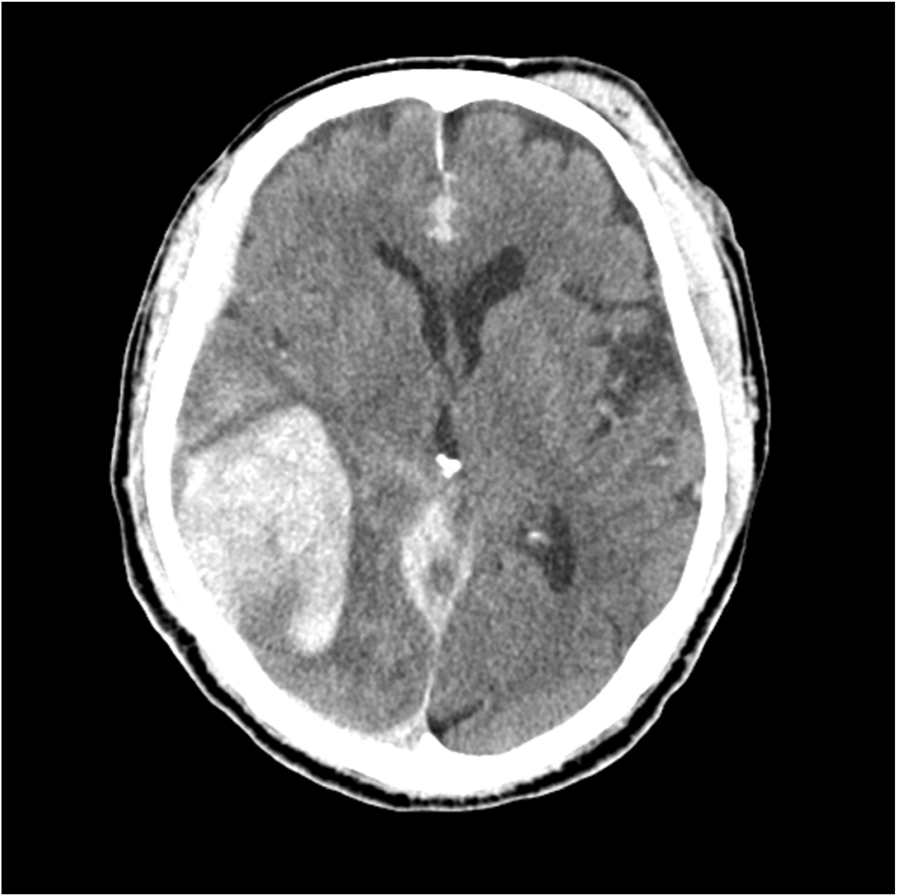
Head CT 3 h after admission showed the expansion of the temporal lobe hematoma

We planned a follow-up CT 3 h later and observed him carefully in the intensive care unit. The second CT showed that the temporal lobe hematoma had increased to 80 × 80 × 40 mm (Fig. 2). At this time (7.5 h after the last taking of dabigatran), his GCS score was 13 (eyes, 3; verbal, 4; motor, 6) and manual muscle test results of 3/5 degrees indicated left hemiplegia. We decided to perform an emergency craniotomy for hematoma removal. At this time, we were informed of his medical history and daily prescriptions by his primary care hospital.

His past medical history included atrial fibrillation, and his daily prescriptions included dabigatran 220 mg. Immediately after learning this, we administered 5 g of idarucizumab by intravenous injection at 5.5 h after the injury (9.5 h after the last taking of dabigatran), and the craniotomy was begun at 6.5 h after the injury. There was no bleeding tendency during surgery, no blood transfusions were required, and the amount of bleeding was small.

**Fig. 3:**
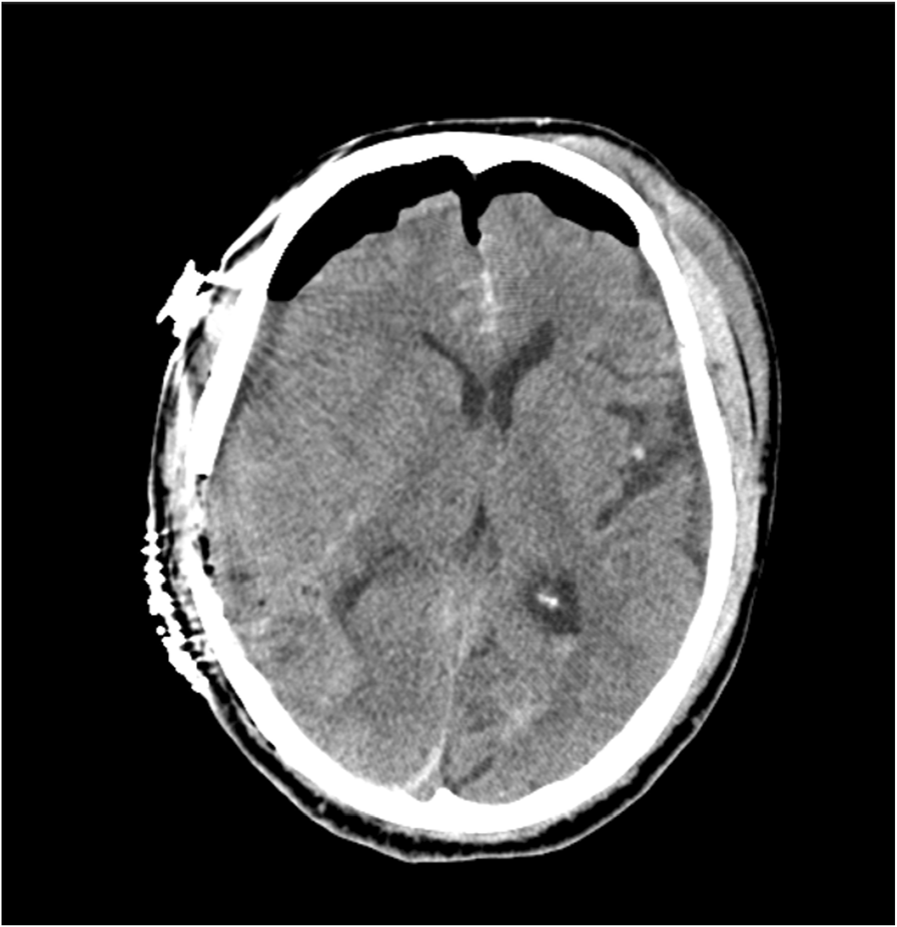
Head CT after surgery showed the removal of the hematoma

**Fig. 4:**
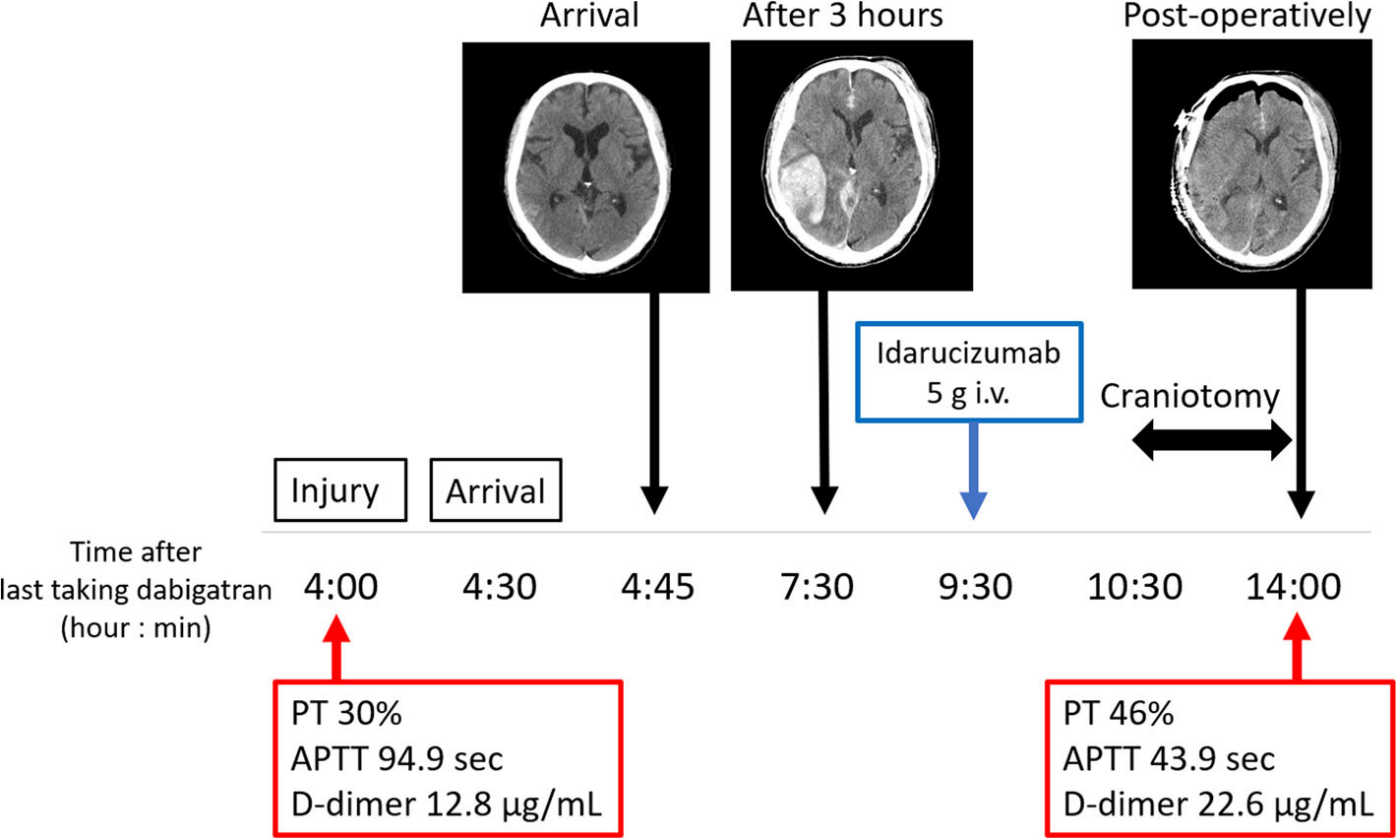
Clinical course, laboratory data, and head CT images

After the surgery, the CT findings revealed that the intracranial hematoma had been removed (Fig. 3), and blood test findings were PT 46%, aPTT 43.9 s, and FDP D-dimer 22.6 μg/mL. The clinical course, laboratory data, and head CT images are shown in Fig. 4. We resumed his dabigatran on postoperative day (POD) 7. A ventriculoperitoneal shunt operation was performed for hydrocephalus on POD 47. He was transferred to a rehabilitation hospital with a Glasgow Outcome Scale score of 3 on POD 102.

### Discussion

Idarucizumab is a humanized monoclonal antibody fragment that binds dabigatran with 350-fold higher affinity than that of dabigatran for thrombin and rapidly reverses its anticoagulant activity. Guidelines for the use of idarucizumab include life-threatening bleeding, bleeding into a critical organ or closed space, prolonged bleeding despite local hemostatic measures, high risk of recurrent bleeding because of overdose or delayed clearance of a drug, and the need for an urgent intervention associated with a high risk of bleeding [8].

Dabigatran has the longest half-life (12–17 h) of any of the DOACs [6, 9]. The dabigatran concentration in the blood in the present patient was thought to be at a high level from the time of injury to the administration of idarucizumab, which likely resulted in the expansion of the intracranial hematoma. It has been recognized that trauma-associated coagulopathy is more common in patients with traumatic brain injury. The coagulopathy results in poor outcomes due to delayed or progressive bleeding and ischemic secondary injury [10–17].

## Conclusion

We present a patient on dabigatran with acute intracranial hematoma who was successfully managed by administering idarucizumab preoperatively. We suggest that idarucizumab could be a potent therapeutic bridge to definitive surgical management in such traumatic brain injury patients taking dabigatran. This is an interesting case that demonstrated the use of idarucizumab in the emergency setting. As more patient populations are exposed to DOACs, reversal medications such as dabigatran may become more prevalent in the emergency department setting, making reports such as this important in laying the groundwork for further research.

## Abbreviations

CT: Computed tomography
DOAC: Direct oral anticoagulants
GCS: Glasgow Coma Scale
POD: Postoperative day
